# Perspectives of medical students on simulation-based training: the Nigerian experience

**DOI:** 10.11604/pamj.2022.43.16.25542

**Published:** 2022-09-08

**Authors:** Chinyere Ezeaka, Iretiola Fajolu, Beatrice Ezenwa, Emeka Chukwu, Shruti Patel, Rachel Umoren

**Affiliations:** 1 College of Medicine, University of Lagos, Lagos, Nigeria,; 2 Department of Pediatrics, University of Washington, Seattle, Washington, United States of America

**Keywords:** Education, paediatrics, medical education, simulation, World Wide Web technology

## Abstract

**Introduction:**

simulation-based education (SBE) is becoming more prevalent in higher education. However, little is known of the perceptions of medical students towards this training approach. The objective of this study was to explore the perceptions of Nigerian medical students on manikin-based and virtual simulation training.

**Methods:**

in January 2019, a paper-based 25-item survey on simulation-based training was administered to a convenience sample of 120 medical students in the 4^th^ year (400 level) and final year (600 level). Data were analysed using descriptive statistics, Pearson´s chi square, and the Fisher´s Exact test.

**Results:**

a total of 95 surveys were completed (RR 79%). Respondents were mostly 21-30 years 95 (81%) and about half were female 60 (51%). Under half of 600 level students 22 (38%) reported receiving simulation-based training in Basic Life Support. A lack of curriculum 27 (28%), instructors trained in simulation education 31 (33%) and funding 52 (55%) were perceived as challenges to manikin-based simulation. Lack of awareness was the greatest single challenge to online simulation 50 (53%). A majority of medical students 181 (96%) owned smartphones, but only 3% (n=3) of respondents had experienced a virtual reality (VR) simulation. If facilities were available, 99% of respondents would recommend the use of online simulation.

**Conclusion:**

there is an opportunity for increased exposure to simulation-based training during undergraduate medical education in Nigeria. Instruction in simulation methods for clinical lecturers in medical schools would increase awareness of the potential advantages of simulation-based training and access for medical students to simulation education.

## Introduction

Simulation education provides medical students with the opportunity to practice clinical skills on a manikin or virtual patient before encountering an actual patient [1-3]. While clinical training has historically relied on practice using real patients, the increased focus on improving the quality and safety of healthcare has led to the increased use of simulation-based education in medical education [4]. While hands-on training with real patients cannot be completely replaced, simulation supports the mastery of fundamental skills while exposing students to different levels of difficulty and rare events, preparing learners to successfully perform potentially dangerous medical procedures.

Simulation-based education has been shown to increase learner proficiency in both technical and non-technical skills [5,6]. Adult learning theories such as the Ericsson´s deliberate practice theory and the Kolb´s experiential learning theory support the practice of simulation [7,8]. This is because simulation encourages the student to repeat skills or behaviors until they have mastered them [7,9]. The simulation experience then can be reflected on and the skills practiced in simulation transferred to clinical practice [8,10]. Simulations can be conducted in the actual clinical setting in designated locations, e.g. Helping Babies Breathe (HBB) corners in labor and delivery wards or in specially designated locations such as clinical skills laboratories which are set up with training equipment including manikins, hospital beds and supplies to mimic a clinical setting [11].

Compared to didactic lectures, simulation education requires a greater teacher to student ratio, equipment, and dedicated space [4,12,13]. For these reasons, online computer-based or virtual reality (VR) simulations are being used in high income settings to supplement manikin-based training [14,15]. Standardized training programmes common in low-resource settings such as the Essential Newborn Care Curriculum (ENCC) and Helping Babies Survive (HBS) feature hands-on, small group practice sessions and simulation using low-cost manikins [16,17]. However, most of these training programmes are directed towards in-service providers and little is known of the access to and perceptions of simulation-based training in medical students in low-resource settings.

The objective of this study was to explore the access to, and perceived utility of, various simulation modalities by medical students at a tertiary institution with a simulation center in Nigeria.

## Methods

A 25-item cross-sectional survey was created by the investigators (RU, CE) who are simulation research collaborators with questions on access to simulation facilities and perceptions on simulation-based training. The development of the survey has been previously described in the context of use for a national survey of paediatric healthcare workers in Nigeria [18]. The study was approved as exempt by the University of Washington Institutional Review Board and ethics approval in Nigeria was obtained from the University of Lagos Health Research Ethics Committee.

**Participants:** in January 2019, the anonymous survey was administered on paper to a convenience sample of 120 medical students at the College of Medicine, University of Lagos, Lagos, Nigeria. All students at the 400 and 600 levels were eligible to participate in the anonymous survey.

### Measures

***Access to simulation-based training facilities:*** respondents were asked questions on their access to simulation-based training facilities, for example: “Does your institution/health facility have facilities for simulation-based training” and “Does your center have a skills-based simulation lab?”. Response options were yes or no. Respondents were asked “In what capacity does your institution use simulation-based training?” Respondents could select from three options which were not mutually exclusive: teaching, research or examination.

***Exposure to simulation-based training:*** respondents were asked about their awareness of and exposure to simulation-based training modalities including manikin-based, online, and virtual reality simulation. Response options were manikin-based or online training with Helping Babies Breathe (HBB), Pediatric Advanced Life Support (PALS), Essential Newborn Care (ENCC), Basic Life Support (BLS), Neonatal Resuscitation Program eSIM, HeartCode (PALS Online course), Online BLS, and Online ACLS course.

***Challenges to simulation-based training:*** respondents were asked questions on the challenges to having a skills-based simulation lab at their center and the challenges to online (computer-based or virtual reality) simulation. Response options on the challenges to having a skills-based simulation lab were lack of funding, lack of access to equipment, lack of curriculum, lack of space, lack of instructors trained in simulation education, and lack of awareness of an option for simulation-based training.

***Perceptions of simulation-based training:*** respondents were asked to identify the advantages of simulation-based training that they were aware of with response options: skills acquisition, provides feedback, step down training, monitoring and evaluation, debriefing/reflection, hands-on skills practice, teamwork training, skills maintenance/retention, and examination purposes when patients are unavailable. Finally, respondents were asked whether if all facilities were available, they would recommend online simulation for their centre with response options: yes or no.

**Data analysis:** data were analysed using descriptive statistics and the Fisher´s Exact test to examine the relationship between training level and respondents´ access and exposure to simulation-based training facilities in their institution or healthcare facility as well as their perceptions of the benefits and challenges in using simulation-based training in their facility. No power calculation or sample size calculation was performed as the sample size was fixed. SAS 9.4 software [SAS Institute, Cary NC] was used for the analysis.

## Results

A total of 95 surveys were completed by 400 level 37 (39%) and 600 level 58 (61%) students (RR 79%). The majority of respondents were 21-30 years of age 95 (81%) with female students making up 51% (n=60) of respondents.

**Figure 1:**
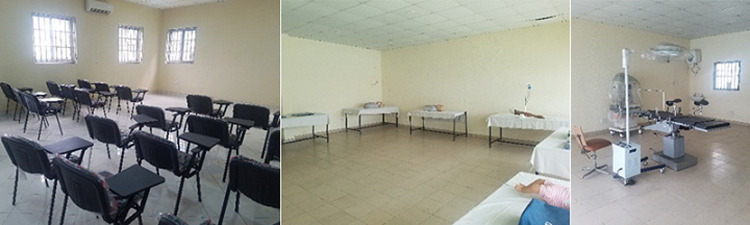
College of Medicine, University of Lagos Simulation Centre

**Simulation-based training facilities:** the simulation skills lab at the University of Lagos has 3 skills rooms and 2 training rooms. The lab is in the process of being set up for full use by the College but is currently equipped with an Operation Room (OR) table, demonstration tables and manikins (Figure 1).

**Exposure to simulation-based training:** the students reported use of simulation facilities for teaching 9 (9%), examination 8 (8%) and/or research 3 (3%) was low. Only 11% (n=4) of 400 level students and 21% (n=12) of 600 level students were aware of the facilities for simulation-based training. One-quarter of the 600 level students had been trained in Basic Life Support (22, 38%), p<0.001. Although 95% (n=90) medical students owned smartphones and 35% (n=33) were aware of VR simulation training, only 3% (n=3) of all respondents had experienced an online or VR simulation.

**Figure 2:**
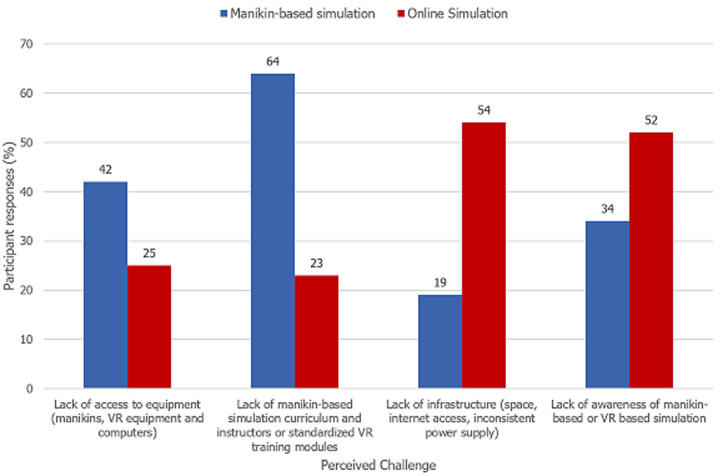
perceived challenges to manikin-based versus online simulation

**Challenges to simulation-based training:** respondents identified challenges to having a skills-based simulation lab and to online (computer-based or virtual reality) simulation (Figure 2). A lack of curriculum 27 (28%), instructors trained in simulation education 31 (33%) and funding 52 (55%) were perceived as challenges to manikin-based simulation in skills-based simulation labs. Lack of awareness was the greatest single challenge to online simulation 50 (53%) but lack of infrastructure [inconsistent power supply 30 (32%) and internet access 23 (24%)] also pose significant challenges.

**Perceptions of simulation-based training:** most respondents identified the advantages of simulation-based training to include skills acquisition 75 (79%), hands-on skills practice 50 (53%), and examination purposes when patients are unavailable 49 (52%). More 600 level students were likely to identify providing feedback (23 (40%); 600 level student vs. 5 (14%); 400 level; p<0.05), skills acquisition (50 (86%); 600 level vs. 25 (68%); 400 level; p<0.05) and hands-on skills practice (36 (62%); 600 level vs. 14 (38%); 400 level) as an advantage of simulation-based training. Few respondents identified debriefing/reflection or step-down training as advantages of simulation-based training (Table 1).

**Table 1:** comparison of perceived advantages of simulation-based training for 400 versus 600 level students

Advantages of simulation-based training	400 level students n (%)	600 level students n (%)	p-value
Skills acquisition	25 (68)	50 (86)	0.04
Provides feedback	5 (14)	23 (40)	0.01
Step down training	1 (3)	7 (12)	0.14
Monitoring and evaluation	13 (35)	23 (40)	0.83
Debriefing/reflection	2 (5)	11 (19)	0.07
Hands-on skills practice	14 (38)	36 (62)	0.03
Teamwork/communication training	9 (24)	25 (43)	0.08
Skills maintenance/retention	15 (41)	30 (52)	0.30
Examination purposes when patients are unavailable	16 (43)	33 (57)	0.21

Ninety-eight percent of respondents thought that simulation-based training could be expanded beyond the current scope with expanded use of simulation for either continued practice after initial training 72 (76%), teaching 71 (75%), and/or research 56 (59%). If facilities were available, 99% of respondents would recommend the use of online simulation for their center.

## Discussion

In this study, we explored the access to and perceived utility of various simulation modalities by learners in a resource-scarce setting. Our study found that lack of awareness and trained facilitators are significant barriers to simulation-based training for medical students, even when a simulation lab is available. This is in contrast with the use of simulation facilities in high resource settings [19,20]. There was a significant interest shown by respondents in greater access to simulation-based training in general, and online simulation in particular.

The access of healthcare learners to simulation-based training has been underreported in low and middle income countries. We found that there is a lack of utilization of skills-based simulation labs in medical student training. This is in contrast with the exposure of post-graduate medical training and in-service healthcare workers, particularly in high income countries [5,21,22]. The majority of paediatric simulation-based training in both low and high income countries is associated with standardized resuscitation training programs such as the Helping Babies Breathe (HBB), Neonatal Resuscitation Program (NRP) and Pediatric Advanced Life Support (PALS) [23,24]. Hybrid training approaches that utilize online simulation (NRP eSIM and HeartCode) and manikin-based simulation are increasingly used in high income countries, particularly with new recommendations for social distancing and restrictions on class sizes [14].

The perceived challenges to establishing skills-based and online simulation training were similar with lack of awareness and access to infrastructure including equipment, consistent power supply and internet access serving as the greatest barriers. Where simulation centers and equipment are available, faculty development in simulation education is also needed to facilitate the local development of simulation training curricula [5,24]. Where trained simulation facilitators are unavailable, remote facilitation can be considered for simulation and debriefing [19].

Medical students surveyed were open to the expansion of simulation for teaching, continuing education and research; and supported the introduction of online simulation. Online simulation is feasible in low resource settings through the widespread availability of mobile phones [20]. We confirmed a high percentage of smartphone use among medical personnel in our study. Although some concern was expressed by participants about lack of internet access or inconsistent power supply as barriers to online simulations, the greatest single challenge identified was lack of awareness. The integration of simulation-based training into medical school curricula at the 400 level would provide opportunities for early and progressive exposure to simulation training.

This study had some limitations. The study was conducted at a single institution and could be subject to response and recall bias. The study design was cross-sectional and could have missed evolving perspectives over time. While medical students were well represented in this survey, other health professional students may have different perspectives and access to simulation-based education and this could be a subject for future study.

## Conclusion

There is a need to expand access to simulation-based education at all levels of medical training. Collaboration between public institutions and private industry can lead to the establishment of Centres of Excellence for Simulation Education. These centres would support both simulation facilitator training and instruction of medical students using various simulation modalities including manikin-based and virtual simulation to increase access to simulation-based education.

### What is known about this topic

- Simulation-based education provides learners the ability to practice and master essential skills while maintaining high quality and safety standards of healthcare practices;
- The efficacy and flexibility of simulation education have been documented in the context of in-service providers, though the perceptions and experiences of medical students towards these programmes remain left to be explored.

### What this study adds

- Medical students are supportive of expanding simulation for educative purposes, particularly online simulation;
- The greatest barriers to simulation training are lack of awareness and trained facilitators.
